# In Vitro Characterization of Agonist and Antagonist Peptide Binding Interaction Kinetics to GLP-1R in HEK293T Cells Using Surface Plasmon Resonance Microscopy

**DOI:** 10.1021/acsmedchemlett.6c00091

**Published:** 2026-05-04

**Authors:** Miyuki A Thirumurthy, Jesús S. Aguilar Díaz de león, Shuchong Pan, Nguyen Ly

**Affiliations:** † Biosensing Instrument Inc., Tempe, Arizona 85284, United States; ‡ Mayo Clinic, Rochester, Minnesota 55905, United States

**Keywords:** GLP-1 receptor, Peptide agonists, GPCR binding kinetics, Surface plasmon resonance microscopy, Extracellular domain, Transmembrane domain coupling, Agonist−antagonist selectivity

## Abstract

The glucagon peptide 1 receptor (GLP-1R), a class-B-type G protein-coupled receptor (GPCR), is a key therapeutic target for many metabolic disorders, including obesity and type 2 diabetes, due to its central role in glucose homeostasis and insulin secretion. Despite its pharmacological importance, studying the binding kinetics of its multidomain engagement with peptide ligands remains a challenge using purified receptor systems. The isolated forms fail to capture the dynamic behavior of membrane-bound GPCRs in a physiologically relevant context. A deeper understanding of the interaction kinetics of agonist and antagonist binding to GLP-1R domains is essential for rational drug design, as the activation of the receptor depends on distinct binding modes that modulate downstream signaling efficacy. Here we employ surface plasmon resonance microscopy (SPRM) on HEK293T cells overexpressing GLP-1R to visualize and quantify the label-free kinetic interactions of ligands on whole single cells in real time. Using three different agonists (GLP-1, liraglutide, exendin-4) and one antagonist (exendin-9), we demonstrate that the agonists exhibit a two-mode/bivalent binding behavior with C-terminal engagement of the extracellular domain (ECD) and N-terminal engagement of the transmembrane domain (TMD). In contrast, the antagonist exendin-9 binds with a single mode, exclusively to the ECD. Importantly, SPRM resolves not only the presence of dual-domain engagement but also the stability and heterogeneity of these interactions, enabling discrimination between full and partial agonism. Notably, liraglutide displays the highest interaction affinity and the greatest amount of activation through TMD binding, which agrees with its known structural optimization and superior therapeutic performance. This study highlights SPRM as a powerful, label-free platform for probing the on-cell binding kinetics of GPCR interactions with peptides, providing quantitative insights into the activation efficiency of agonists and selectivity of antagonists in ways conventional receptor assays cannot.

## Graphical abstract

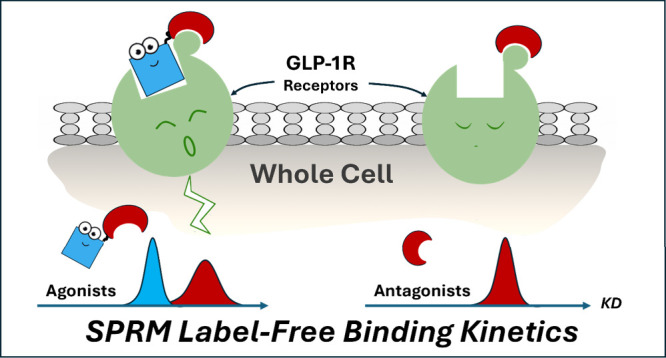

G protein-coupled receptors (GPCRs) form the most common family of membrane receptors which mediate cellular responses to a range of extracellular signals like hormones, peptides and small molecule neurotransmitters.^1^ Because of their critical role in signal transduction into intracellular pathways, GPCRs are targets for majority of the FDA-approved drugs.^2^ This superfamily includes a unique subfamily of class B GPCRs, which respond to peptide hormones such as glucagon, calcitonin, parathyroid hormone, and GLP-1.^3,4^ These class B receptors have a large extracellular domain (ECD) for ligand recognition linked to a complex 7-transmembrane domain (7-TMD) that evokes signaling. The complex dual-domain architecture is utilized by bivalent ligands to produce a multistep binding behavior to enhance their efficacy; however, the traditional assays often fail to observe this complex binding behavior.^5^

Recently, one of the receptors in this family, glucagon peptide 1 receptor (GLP-1R), has gained considerable interest due to its critical functions in glucose homeostasis and energy balance.^6^ Its activation has been at the core of the treatment of type 2 diabetes, obesity, and various cardiovascular disorders.^7^ Despite clinical efficiency, the molecular mechanisms governing the peptide interactions with the receptor remain poorly understood, particularly in the native cell context, where the influence of the receptor conformation and lipid environment strongly impact the kinetics and overall efficacy of a drug molecule.

Being a multidomain membrane receptor, GLP-1R has a strong reliance on its lipid microenvironment, which plays a major role in stabilizing the overall conformation of the receptor. The receptor can accommodate two distinct modes of ligand binding, which involves an initial binding to the ECD, followed by binding to the TMD of the receptor.^8^ In most cases, the agonist–receptor interaction will result in activation of the receptor by promoting a structural change such that it is able to couple to the G proteins. On the other hand, antagonists either favor inactive conformation or inhibit ligand binding to the receptor site. The differential binding kinetics of agonists and antagonists to GLP-1R play a critical role in elucidating the mechanism by which ligand binding leads to various functional outcomes.^9^

Detailed kinetic characterization of agonist and antagonist binding to the GLP-1R can reveal how subtle differences in binding kinetics can govern the strength and duration of receptor activation.^10^ These crucial insights are essential for designing therapeutics with improved pharmacodynamic profiles. Also, their well-characterized clinical and preclinical profiles make them good reference molecules for comparing new GLP-based drugs.

GLP-1R can interact with a wide range of peptide ligands with different efficacy and signaling bias. Liraglutide, a long-acting GLP-1 derivative with modifications at the N-terminus along with a C16 fatty acid side chain, displays sustained receptor activation by binding albumin and having lower clearance rates.^11^ Exendin-4, a peptide isolated from *Heloderma suspectrum* venom, is a well-known potent agonist with enhanced stability to proteolytic degradation.^12^ In contrast, exendin-9, a truncated form of exendin-4, lacks the N-terminal activating domain and acts as a competitive antagonist that binds the receptor without triggering activation.^13^ Comparison of these ligands offers a useful framework for understanding how structural variations alter kinetic behavior and receptor engagement.

Traditional approaches employed to measure the interaction between receptors and ligands, such as radioligand displacement binding assays, are still the most widely employed in the case of GPCRs.^14^ These approaches give very accurate measures of affinity, but the binding characteristics obtained by these methods are averaged values and do not describe the heterogeneous nature of the receptor–ligand interaction in living cells. Biophysical methods such as surface plasmon resonance (SPR) and isothermal titration calorimetry (ITC) offer highly accurate information on kinetic and thermodynamic characteristics, respectively, but are not frequently used for full-length GPCRs because of difficulties involved in preserving their proper folding and functionality under conditions other than that within the natural lipid bilayer.^15,16^ Optical techniques such as fluorescence resonance energy transfer (FRET) or bioluminescence resonance energy transfer (BRET) can be employed to measure interactions in living cells, but these assays are indirect and often detect molecular proximity or changes in signaling activity rather than heterogeneities in the binding kinetics of the receptors. Thus, the development of new label-free technologies that can directly measure the interaction kinetics under physiological conditions becomes necessary. Studying ligand engagement under physiological conditions preserves all natural post-translational modifications and the natural dynamics of conformational changes in multidomain receptors.^17^

Surface plasmon resonance microscopy (SPRM) has emerged as a powerful label-free technique to quantify binding kinetics at the single-cell level.^18–23^ By combining the high sensitivity of SPR with high-resolution imaging capabilities, SPRM enables real-time measurement of molecular interactions at the cell surface without disrupting cell integrity. In this study, SPRM was used to characterize the binding behavior of peptide agonists (GLP-1, liraglutide, and exendin-4) and an antagonist (exendin-9) on HEK293T cells overexpressing GLP-1R. The ability to measure the binding heterogeneity of the peptide–receptor interactions within the cell environment reveals distinct kinetic modes corresponding to mono- and bivalent binding interactions. The findings herein present new mechanistic insight into how structural variations among the GLP-1R peptide ligands govern the binding kinetics and efficacy, providing a strong framework for the rational design of next-generation therapeutics.

## High-Resolution Kinetics Analysis of GLP-1R Binding Interactions on Whole Cells

To study the binding interactions of four different peptide ligands with GLP-1R overexpressed on the surface of HEK293 cells, HEK293 cells were grown and fixed on sensor chips as described in Materials and Methods (Figure 1a). After performing a kinetic titration injection series (Figure 1b) of each peptide on HEK293 cells overexpressing GLP-1R (see Materials and Methods), the data were analyzed as described in Experimental Method and Data Analysis. Briefly, high-resolution kinetic analysis was performed using a virtual grid of 600 regions of interest (ROIs) being applied across the sensor surface. Each ROI was fit to a 1:2 heterogeneous kinetic binding model for the agonists and a 1:1 kinetic binding model for the antagonist, and the extracted kinetic parameters were plotted in an isoaffinity scatter plot. The isoaffinity scatter plot is a 3D representation of the binding interaction behavior. With *k*_d_ plotted along the *x* axis and *k*_a_ along the *y* axis, it facilitates detailed inspection of the binding heterogeneity and statistical analysis of the binding kinetics. Histograms extracted from the isoaffinity plot were fitted with Gaussian distributions to obtain mean values and standard deviations for all the three kinetic parameters: *k*_a_, *k*_d_ and *K*_D_ (taken along with the plot diagonal). Scatter points from the isoaffinity scatter plot also correspond to red ROIs located on the SPR activity maps, with each indicating a localized area of observed binding activity (Figure 2). The confluency of the cells attached to the sensor surface is readily detected by SPR and highlighted in green on the SPR activity maps. The high degree of overlap between the green confluency regions and red ROI activity areas revealed high amounts of cell-specific binding activity for each peptide on overexpressed HEK293 cells and negligible nonspecific binding on areas with no cells.

**Figure 1:**
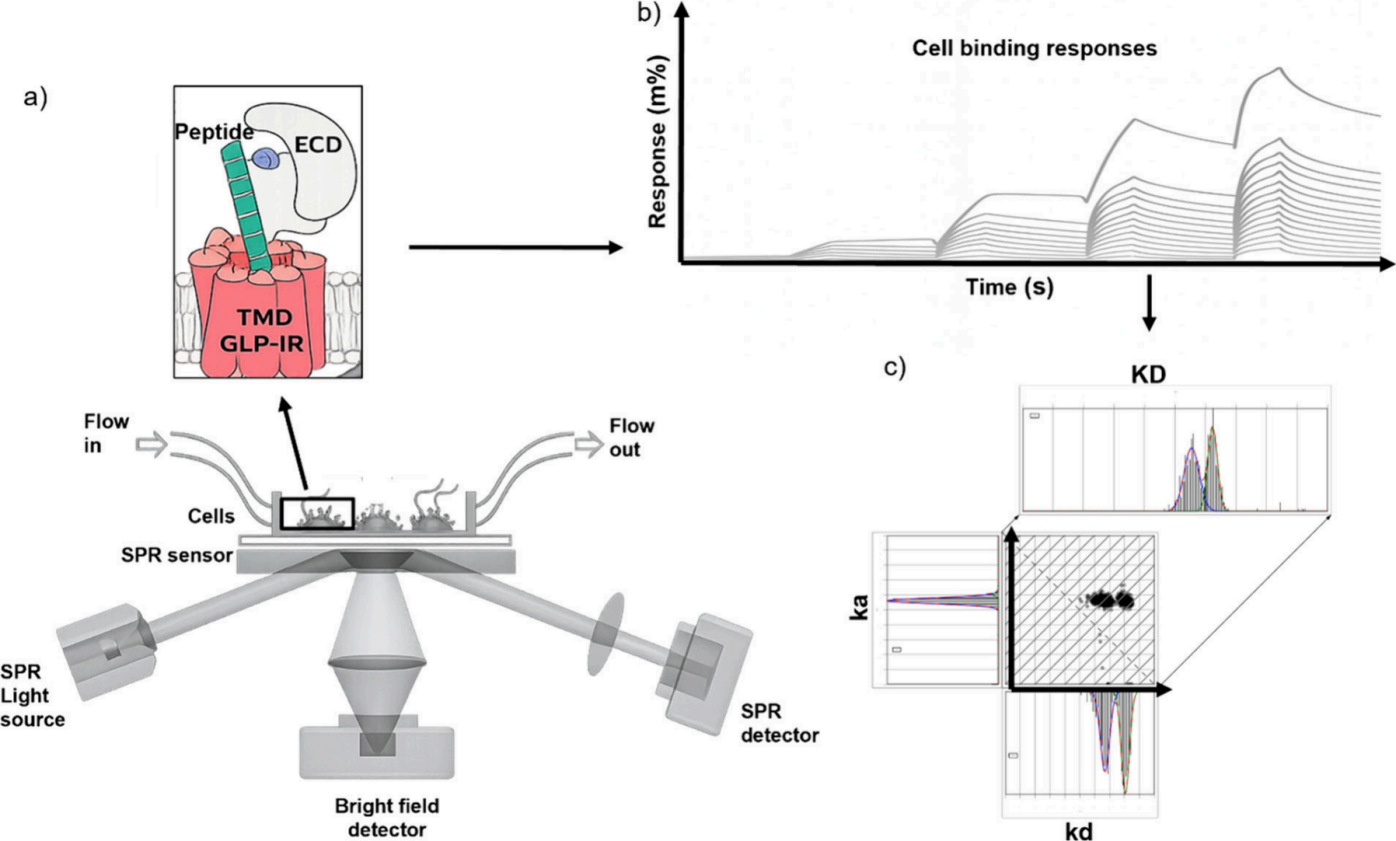
Measuring peptide binding interactions on HEK293 cells expressing GLP-1R using SPRM. (a) Schematic of the SPRM setup. The SPRM light source induces SPR on sensor chips in the presence of adherent HEK293 cells. The plasmonic resonance condition, which is extremely sensitive to changes in dielectric constant due to molecular binding, is recorded by the SPRM detector. Simultaneous bright-field (BF) imaging of the detection area helps to convey cell morphology, confluency, and other phenotypes. Peptides are delivered to the cells on the sensor surface via an automated microfluidics system through a flow cell, supporting simultaneous SPR and BF imaging. (b) The entire SPR sensing area is uniformly divided into a virtual grid of 600 regions of interest (ROIs). Simultaneous responses from all ROIs during the kinetic titration injection series are recorded. Every RIO response series is fitted to a kinetic interaction model to extract kinetic parameters. (c) The calculated rate constants *k*_a_ and *k*_d_ from every ROI’s binding interaction series are collected to form an isoaffinity scatter plot, displaying the heterogeneity of the binding interaction. Gaussian distributions are applied to the histograms along each axis and the diagonal to extract the mean and SD for the three kinetic parameters (*k*_a_, *k*_d_, and *K*_D_).

**Figure 2:**
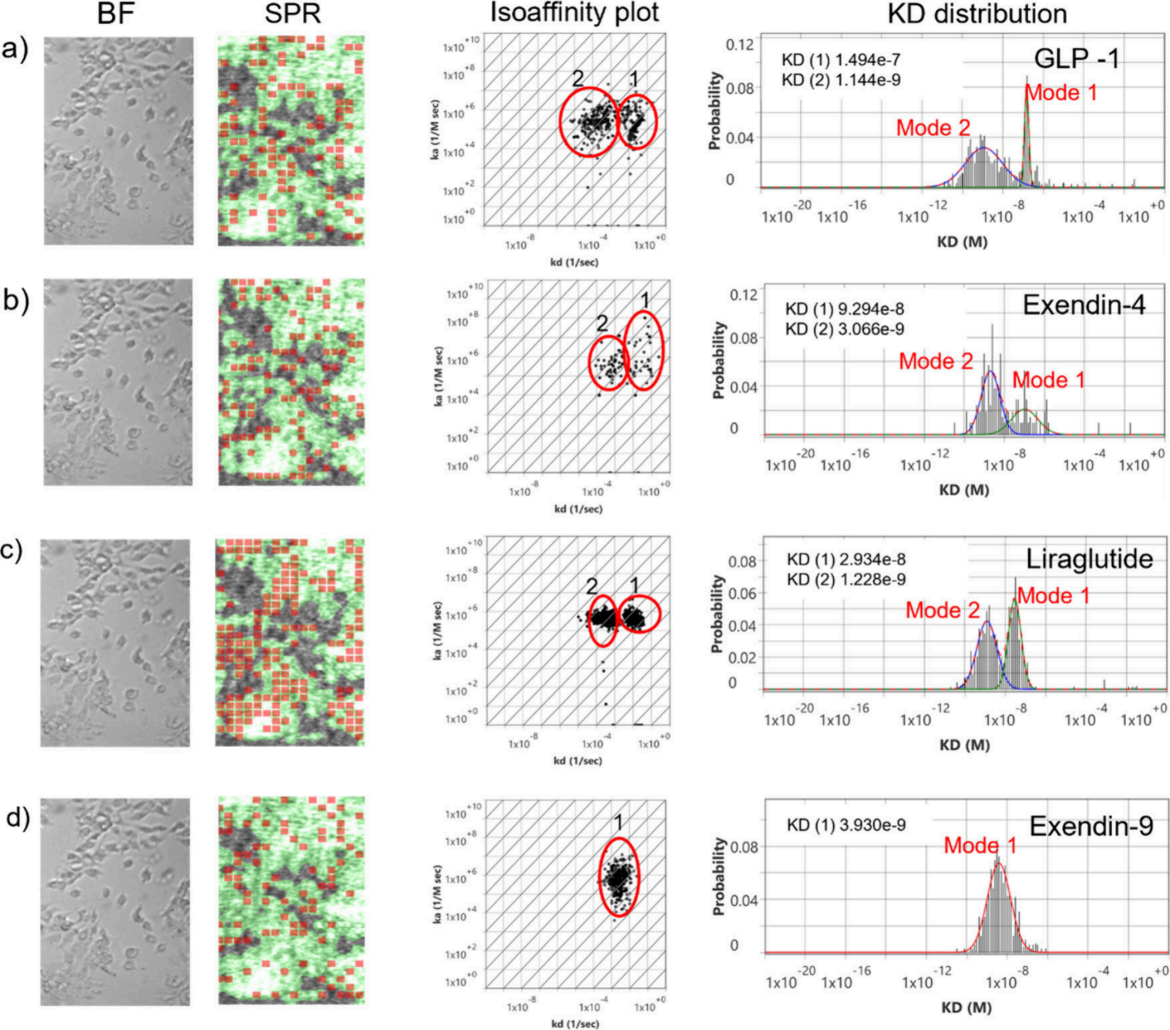
Peptide binding kinetics on GLP-1R-overexpressed HEK293 cell surface. Bright-field images of overexpressed HEK293 cells on the sensor surface and their corresponding SPR images. Red squares are regions of interest (ROIs) that observe responses which closely fit the kinetic binding model. The green regions indicate areas confluent with cells. Active areas designated by red ROIs overlap closely with cell regions indicating high cell specificity and low nonspecific binding. The measured interactions of (a) GLP-1, (b) exendin-4, (c) liraglutide, and (d) exendin-9 with GLP-1R on the surface of HEK293 cells are presented in isoaffinity scatter plots to reveal binding heterogeneity and predominant modes of interaction. Two predominate binding modes (1 and 2) for the bivalent interaction are observed, showing similar on rates but dissimilar off rates. The *K*_D_ histograms displaying mode (1) and mode (2) were extracted from each isoaffinity scatter plot and fitted with Gaussian distributions to statistically determine the means and distributions of the kinetic parameters.

GLP-1R expression levels were further validated upon completion of each SPRM measurement by orthogonal end-point analysis using fluorescence microscopy by exposing the same sensor chip to fluorescently labeled anti-GLP-1R antibody. Fluorescence analysis (Figure 3) measured the amount of functional surface-bound receptors and produced results consistent with the SPRM activity maps shown in Figure 2. Additionally, to confirm anti-GLP-1R binding specificity, SPRM experiments and fluorescence microscopy were repeated with the negative control of parental cells. Anti-GLP-1R antibody showed negligible binding on parental HEK293T cells from both SPRM assays (Figure 4) and fluorescence microscopy assays (Figure 3).

**Figure 3:**
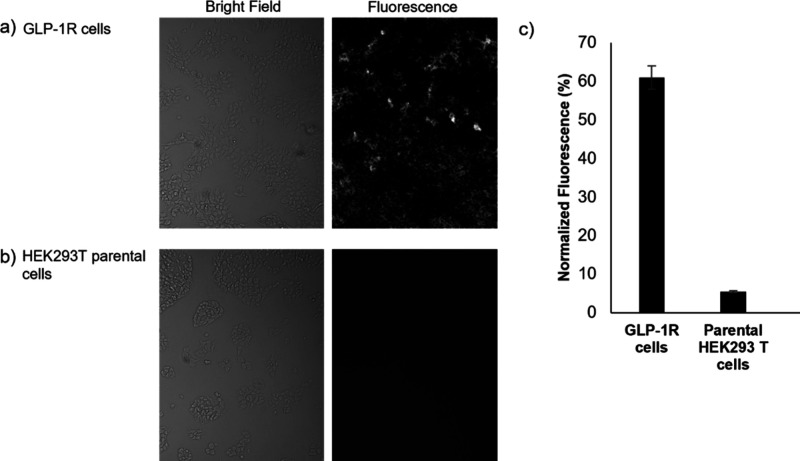
Fluorescence microscopy was performed to verify receptor accessibility. (a) Bright-field and fluorescent images of HEK293 cells overexpressing GLP-1R. (b) Bright-field and fluorescent images of HEK293 parental (negative control) cells showing negligible presence of receptor. (c) Quantification through fluorescence analysis of the relative binding levels of anti-GLP-1R antibody on HEK293 cells overexpressing GLP-1R and parental HEK293T cells. The average fluorescence intensity for each interaction was divided by the total fluorescence from both cell types to give normalized values for comparing relative antibody binding.

**Figure 4:**
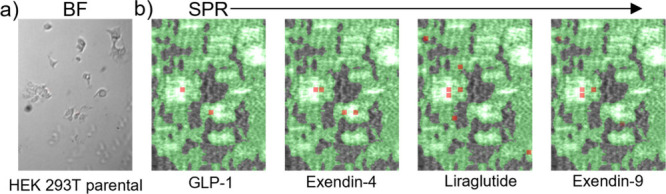
Peptide binding kinetics on parental HEK293T cell surface (negative control). (a) Bright-field image of parental HEK293 cells on the sensor surface and (b) corresponding SPR images. Red squares are regions of interest (ROIs) that observe responses which closely fit the kinetic binding model. The green regions indicate areas confluent with cells. Active areas designated by red ROIs are negligible, indicating the lack of binding to GLP-1R.

## Kinetic Differentiation of Agonist and Antagonist Binding to GLP-1R

In this study, binding affinity (*K*_D_) is used to describe the overall strength of the ligand–receptor interaction, with lower values indicating tighter binding. The interactions of agonists GLP-1, exendin-4, and liraglutide each displayed two distinct kinetic binding modes, as can be observed in their respective isoaffinity plots (Figure 2). The GLP-1 interactions produced *K*_D_ values of 1.1 nM and 149 nM (*k*_a_ values of 2.8 × 10^5^ M^–1^ s^–1^ and 2.5 × 10^5^ M^–1^ s^–1^ and *k*_d_ values of 2.2 × 10^–4^ s^–1^ and 2.4 × 10^–2^ s^–1^, respectively) (Figure 2a). Exendin-4 interactions produced *K*_D_ values of 3 nM and 92 nM (*k*_a_ values of 6.2 × 10^4^ M^–1^ s^–1^ and 1.1 × 10^5^ M^–1^ s^–1^ and *k*_d_ values of 2.2 × 10^–4^ s^–1^ and 2.4 × 10^–2^ s^–1^, respectively) (Figure 2b). Liraglutide interactions produced *K*_D_ values of 1.2 nM and 29 nM (*k*_a_ values of 3.9 × 10^5^ M^–1^ s^–1^ and 1.0 × 10^6^ M^–1^ s^–1^ and *k*_d_ values of 4.8 × 10^–4^ s^–1^ and 1.1 × 10^–2^ s^–1^, respectively) (Figure 2c).

Additionally, as can be readily observed in the isoaffinity plots, the two discrete modes of kinetic binding interactions produced by all three agonists have similar on rates but dissimilar off rates. This distinct binding behavior is representative of avidity-type interactions that can occur with bivalent compounds.^23^ In the case of these agonist peptides, the lower-affinity interaction mode (1) is attributed to single-arm binding of the C-terminus to the ECD domain. This initial interaction facilitates alignment of the ligand for a possible second stage interaction by the N-terminus to the TMD domain (Figure 5). If TMD binding does not occur, then the ligand dissociates, resulting in only the mode (1) interaction occurring. Alternatively, if successive binding to the TMD domain indeed occurs, then the *k*_a_ value for mode (2) remains unchanged from that of mode (1), as the ligand is effectively already bound to the receptor complex. However, the rate of dissociation for mode (2) decreases significantly due to its two-arm attachment condition with both the ECD and TMD regions, resulting in a more stable higher-affinity interaction mode. It is important to note that these two binding modes are mechanistically different from the low- and high-affinity states frequently associated with G protein coupling, as the SPRM measurements here capture ligand–receptor binding interactions at the cell surface without probing downstream signaling complexes or G protein-stabilized conformations. Among the three peptides, liraglutide showed the strongest overall affinity, with a low dissociation rate supporting its extended receptor occupancy and prolonged clinical action compared to the native GLP-1 peptide.

**Figure 5:**
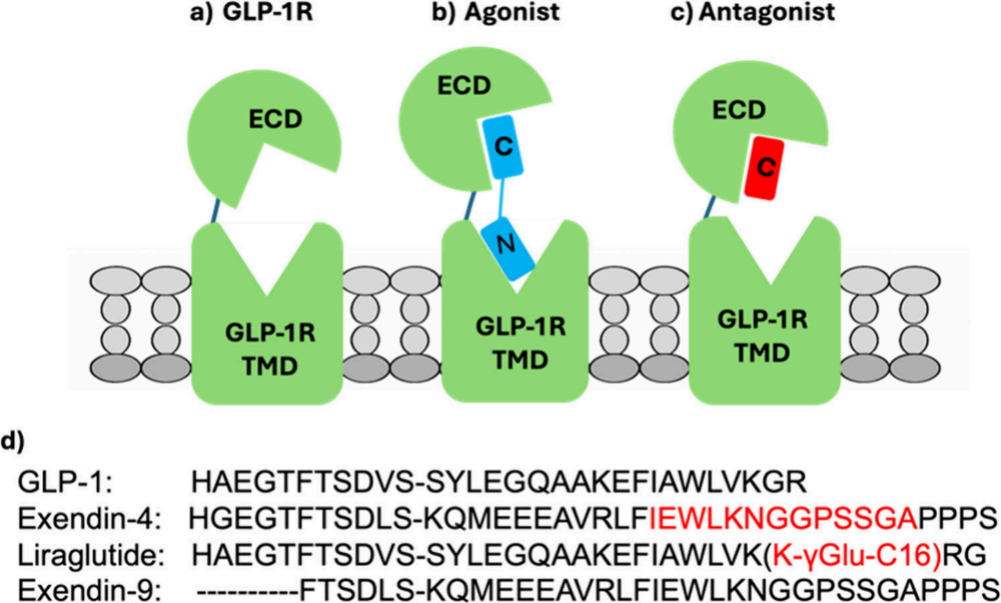
Binding schematic of peptides to GLP-1R. (a–c) Schematic of GLP-1R receptor (a) unoccupied, (b) occupied by an agonist (GLP-1, exendin-4, liraglutide), and (c) occupied by antagonist (exendin-9), illustrating differential engagement of the extracellular domain (ECD) and transmembrane domain (TMD). Agonists bind bivalently, with an initial C-terminal anchoring at the ECD followed by N-terminal insertion into the TMD, whereas the antagonist exendin-9 can only bind to the ECD, effectively inhibiting TMD engagement opportunities. (d) Aligned peptide sequences highlighting structural features that underlie differences in binding kinetics: the rigid Trp-cage motif in exendin-4 (red) that destabilizes TMD engagement and the C16 acylation in liraglutide (red) that enhances membrane residence and N-terminal activation.

In contrast, exendin-9 antagonist interactions produced a single peak with *K*_D_ = 3.9 nM, *k*_a_ = 1.6 × 10^6^ M^–1^ s^–1^, and *k*_d_ = 2.06 × 10^–3^ s^–1^, which are in close agreement with published results^25^ (Figure 2d). Here, a single Gaussian distribution in the *K*_D_ histogram reflects a single mode of interaction of the exendin-9 antagonist with the GLP-1R receptor (Table 1). This behavior is consistent with its monovalent structure, containing only a single binding motif located at the C-terminus, which is intended for binding to the ECD. Exendin-9 thereby aligns with its functional role as a competitive antagonist by stabilizing an ECD-bound receptor conformation without initiating TMD engagement or downstream activation^8,26–28^ (Figure 5).

**Table 1:** Kinetics of Peptide Binding Interactions to In Vitro Membrane-Bound GLP-1R

name	type	mode	*K*_D_ (nM)	*k*_a_ (M^–1^ s^–1^)	*k*_d_ (s^–1^)
GLP-1	agonist	(1)	149	2.53 × 10^5^	2.43 × 10^–2^
		(2)	1.14	2.81 × 10^5^	2.21 × 10^–4^
exendin-4	agonist	(1)	92.9	1.11 × 10^5^	1.22 × 10^–2^
		(2)	3.06	6.22 × 10^4^	5.32 × 10^–4^
liraglutide	agonist	(1)	29.3	1.05 × 10^6^	1.13 × 10^–2^
		(2)	1.22	3.92 × 10^5^	4.81 × 10^–4^
exendin-9	antagonist	(1)	3.93	1.64 × 10^6^	2.09 × 10^–3^

This first of its kind kinetics-based observation of the dual-mode binding behavior of agonists is in agreement with previous biochemical and structural studies of GLP-1R and other class B GPCRs, which demonstrate that the N-terminal region of peptide agonists is essential for TMD engagement and receptor activation, while antagonists lack an N-terminal activation segment.^29,30^ In these studies, N-terminal truncation, point mutants, or peptide chimeras dramatically reduce cAMP signaling while leaving ECD binding largely intact, suggesting that C-terminal docking at the ECD significantly mediates N-terminus insertion at the receptor’s TMD. Crystal and cryo-EM structures further reinforce this two-domain model where the C-terminal portion of GLP-1-like agonists adopts a cap on the ECD while the N-terminus slips into the TMD to stabilize active-state conformations. Hence, our SPRM kinetics-based finding that the agonists displayed a second, slower-dissociating kinetic mode, fully consistent with this established mechanism of agonists, which can transition from the initial ECD anchoring to TMD engagement.^31,32^

Consequently, SPRM’s cell-based kinetic binding analysis demonstrates measurement of the two-stage bivalent binding interaction of agonists to both the ECD and TMD regions and clearly distinguishes this behavior from that of antagonists, which bind to the ECD alone. This unique ability of SPRM to extract detailed kinetic parameters of multivalent interactions on whole cells enables a novel framework for interpreting the distinct binding profiles of agonists (two interaction modes) and antagonists (one interaction mode) of class B GPCRs.

The accuracy of the SPRM assay in detecting bivalent peptide interactions on whole cells was evaluated by measuring the interassay precision and reproducibility. A total of 12 peptide measurements were run, each on different sensor chips and days and from different cell culture batches, producing an interassay precision (CV) of 14.2%, indicating high robustness and precision for SPRM cell-based kinetic interaction analysis (Table 2).

**Table 2:** SPRM Reproducibility Assay^a^

name	type	*K*_D_ (nM) from three different batches	mean (nM)	SD	CV (%)
GLP-1	agonist	149	149	1.01	0.67
		1.14	1.4	0.19	15.1
GLP-1		148			
		1.46			
GLP-1		150			
		1.13			
exendin-4	agonist	92.9	93.6	2.60	2.70
		3.04	2.55	0.50	19.6
exendin-4		96.3			
		2.05			
exendin-4		91.5			
		2.57			
liraglutide	agonist	29.3	24.5	4.90	20.1
		1.24	1.02	0.19	18.6
liraglutide		19.5			
		0.87			
liraglutide		24.7			
		0.94			
exendin-9	antagonist	3.93	3.29	0.71	21.6
exendin-9		2.53			
exendin-9		3.41			
overall					14.2

a A total of three SPRM experiments were performed on peptides on HEK293 cells overexpressing GLP-1R from three different batches and different sensor chips. *K*_D_ values for each compound were pooled from across all three different experiments to determine the mean, standard deviation (SD), and coefficient of variation (CV).

## Influence of Peptide Structure on Binding Kinetics

Class B GPCR activation proceeds through a two-domain binding mechanism in which the peptide undergoes anchoring to the ECD followed by engagement with the TMD. The kinetically distinct binding modes observed here correspond to structurally defined interaction states which are the ECD-only anchoring mode (1) and dual-domain ECD–TMD engagement mode (2).^33^ To understand the ligand-specific activation competence, the parameters in Table 3 are considered. The cell-specific active ROI count reflects the total number of ROIs observing ligand–receptor interactions, with a greater number indicating more interaction activity. The mode (2) area under the curve (AUC) is a measure of the percentage of observed interactions attributed to mode (2) dual-domain (ECD + TMD) engagement, with a higher value indicating greater effectiveness in the ligand’s ability to successfully transition from mode (1) into mode (2).

**Table 3:** SPRM-Derived Microstate Heterogeneity and Relative Activation Competence of GLP-1R Ligands^a^

ligand	cell-specific active ROIs	mode (2) AUC (%)	mode (1) width σ_1_	mode (2) width σ_2_	activation factor (%)	activated mode(2) ROIs
GLP-1	247	78	0.32	2.38	∼42	∼81
exendin-4	179	64	1.52	1.06	∼94	∼107
liraglutide	323	48	0.63	0.71	∼100	∼203
exendin-9	128	–	0.95	–	–	–

a The cell-specific active region of interest (ROI) count reflects the total number of ligand–receptor interaction sites detected per cell. Mode (2) AUC reports the percentage of interactions successfully transitioning into TMD engagement. Mode-specific distribution widths quantify the heterogeneity of extracellular-domain mode (1) anchoring and dual-domain extracellular and transmembrane domain (ECD + TMD) mode (2) engagement (σ_1_ and σ_2_, respectively). The microstate activation factor is inversely proportional to the standard deviation of the mode (2) distribution width (*f* ∝ 1/σ_2_) and approximates the efficiency of receptor activation at the TMD.^35^ The activated mode (2) ROIs metric is a uniquely powerful metric for making insightful comparisons of activation performance among the agonists. It is the combined product of cell-specific active ROIs, activation factor, and AUC.

Mode-specific distribution widths (σ_1_ and σ_2_) quantify the heterogeneity and stability of ECD anchoring and TMD engagement, with broader distribution widths indicating greater microstate diversity. The heterogeneity observed by SPRM indicates that the receptor may have different conformations rather than being locked into a single bound state.^34,35^ Such ligand-induced differences in receptor-state-dependent conformational entropy have been extensively documented in cryo-EM, NMR, EPR, and MD simulations of various GPCRs and have consistently shown that agonists tend to occupy ensembles of active, intermediate, and weakly active microstates rather than a single activated state.^36–38^ Therefore, all mode (2) microstates may not correspond to successful receptor activation at the TMD, and hence, understanding the extent of agonism from full agonism (most mode (2) microstates are signaling-competent) to partial agonism (a subset of mode (2) microstates are signaling-competent) is critical in evaluating ligand performance.^39^

Toward this effort, an entropy-based approach to approximating receptor-level activation efficiency has been described here, in which the microstate distribution breadth is related to a certain probability of achieving the activation state.^35,37,40–43^ Using this entropy-based metric, the microstate heterogeneity measured by SPRM may be implemented to approximate how efficiently mode (2) interactions are converted into receptor activation at the TMD. Consequently, the microstate activation factor, which is inversely proportional to the standard deviation of the mode (2) distribution width (*f* ∝ 1/σ_2_), approximates the efficiency of receptor activation at the TMD.^35,36,38,44^ Ultimately, the activated mode (2) ROI parameter may be determined, which represents an approximation for the expected number of ROIs activated at the TMD. While this metric does not represent a direct measurement of downstream signaling, it enables quantitative comparison of relative activation efficiency among agonists and offers mechanistic insight into how microstate heterogeneity influences the overall receptor function.

Although GLP-1 exhibits the weakest mode (1) affinity among the agonists (149 nM), it exhibits a high active ROI count (247) and mode (2) AUC (78%), indicating that many ECD-anchored binding events successfully transition into dual-domain engagement (Table 3). However, the broad mode (2) distribution width (σ = 2.38) reveals substantial heterogeneity within that mode, consistent with the presence of known multiple TMD-engaged microstates.^45–47^ GLP-1 exhibits a low activation factor (*f* ≈ 42%), indicating that despite frequent TMD engagement, only a much smaller proportion of these interactions correspond to microstates which can activate the receptor. This low activation efficiency is consistent with GLP-1 acting as a known partial agonist at the microstate level and indicates that the broad mode (2) distribution reflects variable stabilization of distinct TMD conformations rather than uniform and complete receptor activation.

Exendin-4, by comparison, displays a lower overall cell-specific active ROI count of 179. This reduction in activity primarily indicates less overall binding interaction at the cell surface. Consistent with this observation, the broad distribution width of mode (1) (σ = 1.52) indicates heterogeneous ECD anchoring (Table 3). Inefficient ECD anchoring constrains overall population-level receptor engagement, which limits the number of interactions that can successfully propagate into mode (2). From an ensemble perspective, this reduced propagation results in a smaller pool of TMD-engaged microstates, but one that still retains appreciable conformational entropy. This behavior has been reported with the presence of the Trp-cage motif (Figure 5) in exendin peptides, which could impose steric constraints near the C-terminus and limit conformational adaptability at the ECD.^48^ Consistent with this intermediate ensemble behavior, the entropy-based SPRM analysis yields a high activation factor for exendin-4 (*f* ≈ 94%) that lies between GLP-1 and liraglutide, reflecting partial stabilization of microstates. These observations demonstrate that poor structural anchoring at the ECD reduces dual-domain binding, even when the overall binding affinity appears favorable. Importantly, this suggests that the observed binding modes might primarily arise from peptide receptor structural compatibility at the extracellular interface rather than from intracellular G protein-stabilized binding modes.^39,49^

Not surprisingly, liraglutide exhibits the most favorable display of all measured parameters and behaves well as a known full agonist, efficiently coupling TMD engagement to receptor activation. It produces the highest cell-specific active ROI count (323) among the peptides examined. The narrowest of distribution widths observed for both mode (1) (σ = 0.63) and mode (2) (σ = 0.71) reflect robust and less heterogeneous ECD anchoring coupled to stabilized TMD engagements (Table 3). Narrow distributions correspond to reduced conformational entropy and a smaller number of receptor microstates. Structural and computational studies of lipidated GLP-1 analogs have shown that membrane association and reduced peptide flexibility bias the receptor toward a smaller number of signaling-competent conformations.^50^ This SPRM-based entropy model directly measured distribution widths to estimate the fraction of competent microstates, yielding the highest relative activation competence among the study’s ligands (*f* ≈ 100%). These features are consistent with liraglutide’s lipidation-mediated membrane association and optimized peptide architecture (Figure 5), which reduce conformational freedom and promote sustained dual-domain binding.^50^ This kinetic behavior aligns with liraglutide’s optimized in vivo pharmacology, including prolonged plasma half-life (∼11–15 h versus 1–2 min for native GLP-1), once-daily dosing, and superior clinical efficacy observed in head-to-head trials such as LEAD-6 and subsequent meta-analyses.^51–56^

Finally, the antagonist exendin-9 exhibits exclusively mode (1) binding with a moderate distribution width (σ = 0.95), indicating moderate stability. Unlike the agonists, exendin-9 lacks N-terminal residues required for TMD binding and receptor activation (Figure 5). By occupying the ECD binding site without TMD engagement, exendin-9 stabilizes a receptor conformation which is not capable of downstream signaling. This behavior is consistent with its established role as an antagonist, effectively blocking receptor activation while maintaining stable ECD binding. The absence of mode (2) in its kinetic profile further supports the requirement of the N-terminal TMD interaction for productive GLP-1R activation.

## Conclusions

Traditional biochemical assays often lack the resolution needed to distinguish between dual-domain kinetic states, motivating the use of label-free SPRM to enable direct high-resolution kinetic measurement of both agonist-specific two-stage binding and antagonist restricted ECD engagement. In this work, cell-based SPRM provided a high-resolution, real-time view into how four clinically relevant peptides interact with GLP-1R in their native membrane environment. Three agonists (GLP-1, exendin-4, and liraglutide) consistently exhibited two discrete kinetic interaction modes, representing the sequential engagement of the ECD followed by stabilization within the TMD. This two-phase behavior is driven by avidity-like bivalent interactions, which is in stark contrast to the strictly monovalent, single-mode ECD binding exhibited by the antagonist exendin-9. Importantly, although all agonists accessed mode (2) at the TMD, quantitative SPRM analysis revealed pronounced differences in mode (2) interaction kinetics, stability, and activation. Liraglutide displayed the narrowest mode (2) distribution, highest affinity, and greatest activation efficiency, consistent with its behavior as a top-rated full agonist. In contrast, GLP-1 exhibited a markedly broader mode (2) distribution and lowest activation efficiency, indicative of heterogeneous TMD engagement encompassing many nonactivating microstates, consistent with partial agonism. These results establish SPRM as a uniquely powerful platform for examining GPCR peptide interactions on intact cells, where native lipid composition and receptor conformation are preserved.

## Materials and Methods

### Materials

BI cell chamber kits on Au film sensors (cat. no. 104-00228) and an SPRm 220 microscope from Biosensing Instrument Inc. were used for all the experiments. Trypsin-EDTA solution, 1× (cat. no. 30-2101) was purchased from ATCC. Paraformaldehyde (PFA) (4%, cat. no. J61899) and dimethyl sulfoxide (DMSO) (cat. no. J66650 AE) were purchased from Thermo Fisher Scientific. Peptides GLP-1 (cat. no. HY-P0055), liraglutide (cat. no. HY-P0014), exendin-4 (cat. no. HY-13443), and exendin 9 (cat. no. HY-P0264), each with a purity of 95% or higher as determined by HPLC, were purchased from MedChemExpress. Alexa Fluor 488-conjugated anti-GLP-1R antibody (cat. no. FAB28141G-100UG) was purchased from R&D Systems.

### Generation of a Stable GLP-1R Overexpressing Cell Line

Lentiviral production was carried out in a Biosafety Level 2 (BL2) facility. The GLP-1R lentivirus was generated by cotransfecting HEK293T cells with the pLenti-GLP-1R expression plasmid and the appropriate packaging plasmids using Turbofectin transfection reagent (Origene) according to the manufacturer’s instructions. Briefly, HEK293T cells were seeded in 10 cm culture dishes containing high-glucose Dulbecco’s modified Eagle’s medium (DMEM) supplemented with l-glutamine, sodium pyruvate, and 10% fetal bovine serum (FBS). The following day, cells were cotransfected with 5 μg of pLenti-GLP-1R plasmid and 6 μg of packaging plasmids using Turbofectin. After overnight incubation, the transfection medium was replaced with 12 mL of fresh HEK293T culture medium. The lentiviral supernatant was harvested, clarified by centrifugation, filtered through 0.45 μm cellulose acetate filters to remove cellular debris, snap-frozen, and stored at −80 °C until use. HEK293T cells were transduced with the GLP-1R lentivirus in the presence of 5 μL of Polybrene (8 μg/mL) and incubated for 2–3 days. Transduced cells were subsequently selected with puromycin (5 μg/mL) until most surviving cells expressed GFP. Functional validation of GLP-1R expression was confirmed by assessing cyclic AMP (cAMP) production following stimulation with GLP-1 peptides.

### Cell Culture and Cell Seeding

HEK293/human GLP-1R cell line and HEK293T parental cells were kindly provided by S.P. HEK293/human GLP-1R cell line and HEK293T parental cells were cultured in Dulbecco’s modified eagle medium (DMEM) (Gibco, cat. no. 11885-084) supplemented with 10% FBS and 1% penicilllin/streptomycin solution (Gibco, cat. no. 15-140-148). The overexpressed strain was grown in the presence of 10 μg/mL puromycin (Gibco, cat. no. A1113803) for selectivity. Cells were detached using a 0.05% trypsin-EDTA solution (ATCC). All cells were maintained in an incubator at 37 °C with 5% CO_2_ before seeding. For SPRM sensor chips, about 10,000 cells at 99% viability (determined with trypan blue using a BioRad TC20 cell counter) were seeded onto PLL-precoated SPRM sensor chips containing 400 μL of DMEM medium supplemented with 10% FBS and then incubated at 37 °C with 5% CO_2_. After 72 h of incubation, cells were washed with HBSS buffer. Cells were then fixed with 4% paraformaldehyde for 10 min at room temperature and washed three times in HBSS buffer, immediately followed by SPRM analysis. All experiments were performed with commercially available cell lines. As such, institutional review board approval was not required, in accordance with ACS ethical guidelines.

### Surface Plasmon Resonance Microscopy

An SPRm series 220 surface plasmon resonance microscope (Biosensing Instrument Inc., Tempe, Arizona), which has an integrated microscope with simultaneous bright-field imaging and SPR microscopy (Figure 1a), was used for all SPRM experiments. The SPRM is highly sensitive to analyte binding events on the cell membrane, which cause changes in dielectric constant that are recorded as reflectivity changes in sensorgrams. Additionally, the high-resolution diffraction-limited optics enables detailed subcellular evaluation of the binding heterogeneity.

### Fluorescence Microscopy

The overexpressed and parental cells were seeded in sensor chips (Figure 3), fixed, and analyzed by SPRM with serial dilutions of the Alexa 488-conjugated anti-GLP-1R antibody as described above. After every SPRM experiment, sensor chips were evaluated for fluorescence using a homemade fluorescence microscope.

### Experimental Method and Data Analysis

SPRM measurements were generated by segmenting the sensing area (780 μm × 585 μm area) into 600 virtual ROIs. A kinetic titration series of increasing sample concentration by double for eight injections was run for every interaction analysis (Figure 1B). All measurements were conducted under continuous flow at a rate of 100 μL/min. Every injection in the series had an exposure time of 7 min and a rinse time of 5 min. All the data were collected in real time. Top concentrations for peptides targeting GLP-1R were 100 nM. For every ROI, a 1:2 kinetic binding interaction model was applied for the agonists and a 1:1 model was applied for exendin-9, as it does not follow the 1:2 binding mechanism. ROIs on the bare areas were subtracted as references from cell area ROIs.

The SPRM kinetic analysis results of all ROIs were aggregated for statistical analysis. ImageSPR software (Biosensing Instrument Inc., Tempe AZ) was used to generate the sensorgrams, fitting, and statistical analysis of binding interactions. ROIs that detected a response which fitted well to the kinetic interaction model were collectively plotted into an isoaffinity scatter plot for statistical analysis of the binding heterogeneity. Histograms extracted from the isoaffinity scatter plot were fit with Gaussian distributions to obtain mean values and standard deviations for the association rate (*k*_a_), dissociation rate (*k*_d_), and equilibrium dissociation constant (*K*_D_). Subsequently, those ROIs that observe binding responses were highlighted in red and overlaid onto the SPRM, and optical bright-field images to more clearly evaluate the extent of cell-specific and nonspecific binding. All interaction studies were validated with negative control (parental cells) to verify receptor-specific interactions and with an orthogonal end-point analysis fluorescence technique.

### Safety Statement

No unexpected or unusually high safety hazards were encountered.

## Abbreviations

GLP-1R: glucagon peptide 1 receptor
GPCR: G-protein coupled receptor
SPRM: surface plasmon resonance microscopy
TMD: transmembrane domain
ECD: extracellular domain
SPR: surface plasmon resonance
NMR: nuclear magnetic resonance
MD: molecular dynamics
EM: electron microscopy
EPR: electron paramagnetic resonance
AUC: area under the curve.
